# Sensitivity of a Qualitative 5-Element Cortical Sign Screen for Detecting Acute Basilar Artery Occlusion

**DOI:** 10.1016/j.acepjo.2025.100167

**Published:** 2025-05-21

**Authors:** Christiana Agbonghae, Rahul R. Karamchandani, Dale Strong, Tsai-Wei Wang, Sagar Satyanarayana, Hongmei Yang, Jeremy B. Rhoten, Gary Defilipp, Jonathan D. Clemente, Katelynn J. Teli, Andrew W. Asimos

**Affiliations:** 1 Department of Emergency Medicine, Atrium Health’s Carolinas Medical Center, Charlotte, North Carolina, USA; 2 Department of Neurology, Neurosciences Institute, Atrium Health, Charlotte, North Carolina, USA; 3 Quality Division, Clinical Quality Analytics, Atrium Health, Charlotte, North Carolina, USA; 4 Department of Radiology, Atrium Health, Charlotte, North Carolina, USA; 5 Neurosciences Institute, Atrium Health, Charlotte, North Carolina, USA

**Keywords:** basilar artery, coma, ischemic stroke, thrombectomy, emergency medicine

## Abstract

**Objectives:**

Large vessel occlusion stroke screens primarily identify anterior circulation large vessel occlusion ischemic strokes. Our primary objective was to assess the sensitivity of FANG-D, which screens for visual Field deficit, Aphasia, Neglect, Gaze preference, and Dense limb weakness, to detect basilar artery occlusion (BAO).

**Methods:**

We conducted a retrospective study of BAO strokes (May 2018-February 2024) to assess sensitivity of the FANG-D screen to detect acute BAO. BAO site (proximal, mid, or distal) was confirmed by a neuroradiologist, and occlusions were classified as total or subocclusive. FANG-D was performed by the treating physician; National Institutes of Health Stroke Scale Score (NIHSSS) was performed by neurology consultants.

**Results:**

Of 204 patients with BAO identified, 121 had FANG-D documented. Patients without FANG-D had significantly lower Glasgow Coma Scale (GCS) scores (11 [5-15] and 14 [8-15], respectively). Among BAO patients with FANG-D, sensitivity for detecting subocclusive or total occlusive BAO was 81.8% (74.0%-87.7%). FANG-D negative BAO cases had significantly higher GCS scores (15, IQR: 15-15) and lower NIHSSS (3, IQR: 1-4) than FANG-D positive cases (13, IQR: 7-15 and 12, IQR: 5-25, respectively). The sensitivity of NIHSSS ≥ 6 for detecting BAO in all patients with an NIHSSS (n = 197) was 64.2% (57.4%-70.5%).

**Conclusion:**

A qualitative screen composed of cortical signs lacks sufficient sensitivity to be used alone to screen for acute BAO. Our findings support the importance of considering acute BAO in patients presenting with NIHSSS < 6.

**The Bottom Line**

Without prompt recognition and treatment, basilar artery occlusion (BAO) strokes can result in severe disability or death. This study explored how well a stroke screen comprised of the cortical signs associated with carotid and middle cerebral artery occlusion strokes performed in identifying acute BAOs. The screen (FANG-D) is positive if any of the following deficits are present: a visual Field cut, Aphasia, Neglect, Gaze preference, or Dense limb weakness. Our results found FANG-D can miss up to one-quarter of patients with BAO, making it insufficiently sensitive to be used alone to screen for BAOs.

## 1 Introduction

### 1.1 Background

Acute basilar artery occlusion (BAO) is typically associated with severe disability and high mortality without recanalization.^1^, ^2^, ^3^ The benefit of recanalization via thrombectomy for anterior circulation large vessel occlusion (LVO) ischemic stroke has been well-established for almost a decade. Recently, however, the Endovascular Treatment for Acute Basilar Artery Occlusion (ATTENTION) and the Basilar Artery Occlusion Chinese Endovascular (BAOCHE) trials showed a clear benefit of endovascular thrombectomy for BAO stroke in reducing disability and mortality.^4^^,^^5^ Based on these data, prompt recognition of potential BAO is essential to facilitate time-sensitive endovascular treatment.

### 1.2 Importance

Many stroke severity screens have been developed to identify patients with LVO; however, these tools were designed to primarily identify anterior circulation LVO (ACLVO).^6^^,^^7^ We previously demonstrated one such qualitative cortical sign screen (FANG-D, visual Field deficit, Aphasia, Neglect, Gaze preference, Dense limb weakness) was a sensitive test for identifying ACLVO.^8^ Furthermore, it was highly sensitive for identifying cases that underwent thrombectomy, but few BAOs were included.^8^ Additionally, we found substantial interrater reliability for the overall screen results (positive or negative).^8^ Given the basilar artery provides perfusion to the pons, through which the corticospinal tracts traverse, and branches into the posterior cerebral arteries (which perfuse the occipital lobes), we hypothesize FANG-D may be sensitive for detecting BAO because involvement of these regions may produce limb weakness and anopsia,^9^ which are components of the FANG-D screen.

### 1.3 Goals of This Investigation

Our primary objective was to assess the sensitivity of FANG-D to detect acute BAO. Our health care system’s stroke network has amassed a substantial number of cases of acute BAO in a registry. We have collected FANG-D and National Institutes of Health Stroke Scale Score (NIHSSS) data for this population. As many clinicians consider NIHSSS to be the “gold standard” for stroke severity assessment,^10^, ^11^, ^12^ a secondary objective was to evaluate sensitivity of an NIHSSS threshold to detect BAO. Additional secondary objectives included assessing the sensitivity of FANG-D and a thresholded NIHSSS for the subset of totally occlusive BAOs and for BAO cases undergoing mechanical thrombectomy.

## 2 Methods

### 2.1 Study Design and Setting

This is a retrospective observational study using registry data from a prospectively maintained “Code Stroke” registry for a health care system’s regional stroke network. The stroke network includes an urban, academic, comprehensive stroke center and its affiliated regional hospitals and free-standing emergency departments (EDs) covering a population region of approximately 3 million people in North Carolina, United States. Demographic, clinical, radiographic, and outcome data for patients for whom the Code Stroke protocol is activated are entered into the Code Stroke registry by trained data abstractors.

**Figure 1:**
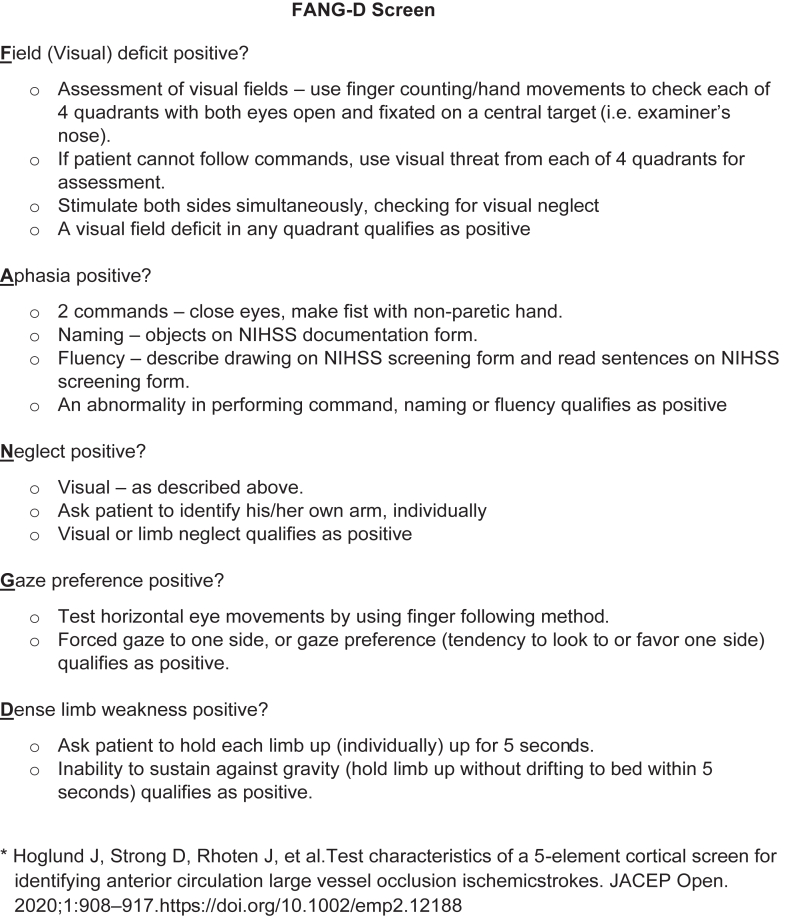
FANG-D screen.^8^ FANG-D, visual Field deficit, Aphasia, Neglect, Gaze preference, Dense limb weakness; NIHSS, National Institutes of Health Stroke Scale.

In April 2018, our stroke network began to routinely use FANG-D as part of the Code Stroke protocol in the ED. Emergency medicine (EM) physicians performed a FANG-D screen on any suspected acute stroke patient presenting within 24 hours of symptom onset, except for patients presenting in an acute comatose-like or locked-in state (a known potential clinical presentation of BAO), for whom FANG-D screen is not necessary.^1^ However, FANG-D was not routinely collected during inpatient Code Stroke activations. EM physicians received training on the FANG-D screen, including the description included as Figure 1.^8^ This, along with an online video with instructions on how to perform the FANG-D screen, were made available for reference for practicing physicians. For patients with FANG-D positive, computed tomography angiography (CTA) of the head and neck is included in the initial Code Stroke imaging. Because the sensitivity of FANG-D for detecting BAO is unknown, our Code Stroke protocol allows for Code Stroke activation and CTA in patients with a FANG-D negative if there is concern for a posterior circulation stroke or BAO based on sudden onset of symptoms referable to the posterior circulation, such as altered mental status with persistent vertigo or ataxia. All FANG-D screens are performed before CTA imaging.

Our institutional review board reviewed our study and determined it met criteria for waiver of authorization and expedited review. This study used the Strengthening the Reporting of Observational Studies in Epidemiology (STROBE) criteria and was approved by our institution’s EM Research Review Committee.

### 2.2 Selection of Subjects

We included 204 patients ≥18 years of age with confirmed total occlusive or subocclusive BAO on CTA imaging, who were treated within our stroke network between May 2018 and February 2024. Patients for whom the Code Stroke protocol was activated in the ED or inpatient setting were included in this study. All CTAs were reviewed by a neuroradiologist to confirm BAO location, which was classified as proximal, mid, or distal based on the following anatomic criteria: proximal = most proximal site of occlusion basilar origin to the anterior inferior cerebellar artery (AICA); mid = most proximal site of occlusion AICA to the superior cerebellar artery (SCA); and distal = most proximal site of occlusion SCA to top of basilar.^13^ BAOs were additionally classified as totally occlusive versus subocclusive. Patients without a confirmed occlusive or subocclusive BAO on CTA imaging were excluded.

### 2.3 Measures

Our primary outcome measure was the sensitivity of the FANG-D screen in detecting total occlusive or subocclusive BAO. FANG-D screens were those documented in the electronic medical record (EMR) by the treating physician. The FANG-D result was classified as positive if any of the 5 elements was present (Fig 1).^8^

We had 3 secondary outcomes: (1) sensitivity of FANG-D for detecting totally occlusive BAOs only (*unlike the primary outcome, which included subocclusive BAOs*), (2) sensitivity of FANG-D for detecting BAO cases undergoing emergent thrombectomy, and (3) sensitivity of NIHSSS ≥ 6 for detecting a BAO overall, and in the 2 subgroups above. Patients with BAO who underwent emergent thrombectomy were identified as an important subgroup by virtue of the fact they underwent acute revascularization therapy. An NIHSSS threshold of ≥6 was chosen based on the recent expert panel recommendation to consider this NIHSSS threshold for thrombectomy candidacy in the setting of BAO.^14^ The NIHSSS used was the first documented score by the consulting neurology team at time of the Code Stroke activation.

The Glasgow Coma Scale (GCS) was abstracted from the nursing assessment section of the EMR at the time of Code Stroke activation. Other demographic, clinical, radiographic, and outcome variables were abstracted from the medical record as described in our Code Stroke Registry REDCap Standard Operating Procedures Data Abstraction and Quality Control document (which includes interrater reliability testing and tracking) and data dictionary. For registry records missing data in the Code Stroke registry and NIHSSS data fields, the EMR was reviewed to obtain these data.

### 2.4 Data Analysis

All statistical analyses were performed using SAS (version 9.4, SAS Institute Inc). Pairwise deletion was employed to minimize the loss of information. Patients with ≥1 data elements not documented were included in this study, but were excluded when performing the calculation. Patients’ demographics, clinical and radiographic characteristics were described using means and SDs for continuous variables, medians and IQRs for ordinal variables, and frequencies and percentages for categorical variables. The Mann-Whitney *U* test was used to compare medians for ordinal variables between groups, whereas the *t* test was used for the mean comparison. χ^2^ tests or Fisher exact tests were used to compare categorical variables. The value of *P* ≤ .05 was defined as statistically significant.

Sensitivity and the associated 95% CIs were calculated for the primary and secondary outcomes. Sensitivity was calculated by dividing the number of “true positives” (eg, individuals correctly identified as having a BAO) by the total number of individuals who had the disease (eg, a BAO, including both true positives and false negatives), expressed as a percentage. The Wilson score interval was used to calculate CIs because it is less sensitive to sample size and effectively handles situations in which the proportion of events approaches 0 or 1 without being overly conservative. We used SAS Proc Freq procedure with binomial (Wilson) option to obtain the point estimate and the 95% CI.

## 3 Results

**Figure 2:**
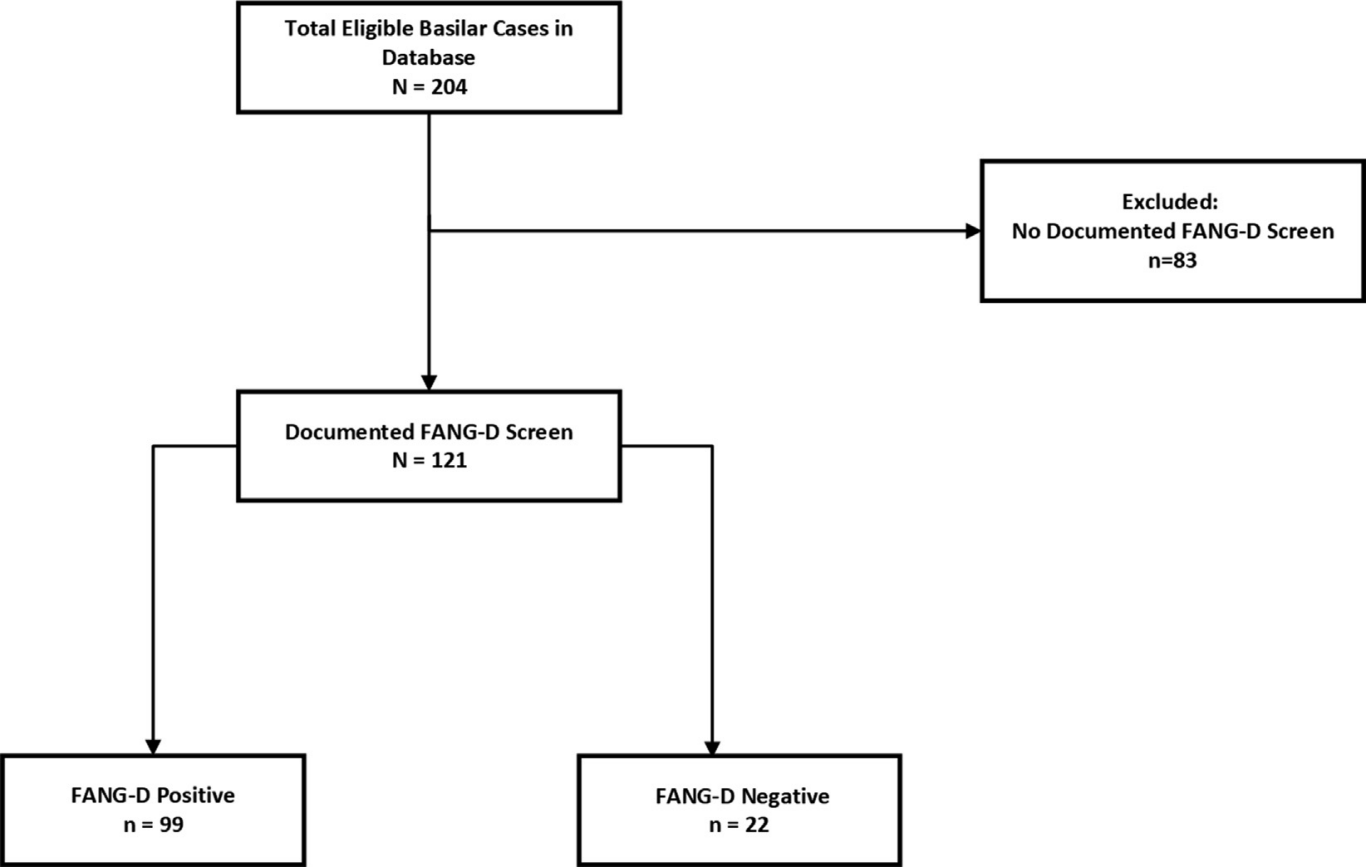
Patient flow diagram. FANG-D, visual Field deficit, Aphasia, Neglect, Gaze preference, Dense limb weakness.

Figure 2 depicts patients included and excluded from this study. As shown in Table 1, mean age of the 204 adult Code Stroke patients with BAO was 67 years (SD, 14) and 60% were male. Eighty-three (40.7%) of 204 patients did not have FANG-D screens. These 83 had significantly lower GCS scores (11, IQR: 5-15) than those with FANG-D recorded (14, IQR: 8-15), *P* < .05. Additionally, inpatient Code Stroke activations were less likely to have a FANG-D screen documented than ED activations (*P* < .001).

**Table 1:** Comparison of patients with and without FANG-D screen results documented.

	Total (N = 204)	FANG-D screen documented (n = 121, 59.3%)	FANG-D screen undocumented (n = 83, 40.7%)	*P* value^a^
Demographics				
Age, y, mean ± SD	67 ± 14	66 ± 15	68 ± 13	.4745
Sex (male), n (%)	123 (60.29)	71 (58.68)	52 (62.65)	.5689
Race (White), n (%)	116 (56.86)	65 (53.72)	51 (61.45)	.2737
Inpatient Code Stroke, n (%)^b^	30 (14.71)	5 (4.13)	25 (30.12)	<.001^d^
Clinical characteristics				
GCS score, median (IQR)	13 (7-15)	14 (8-15)	11 (5-15)	.0217
NIHSS score, median (IQR)^c^	12 (4-23)	9 (4-23)	17 (5.0-26.0)	.0543
Radiographic characteristics				
BAO site				
Proximal, n (%)	68 (33.34)	38 (31.40)	30 (36.14)	.6798
Mid, n (%)	68 (33.34)	43 (35.54)	25 (30.12)	
Distal, n (%)	68 (33.34)	40 (33.06)	28 (33.73)	
Totally occlusive BAOs	157 (76.96)	90 (74.38)	67 (80.72)	.2906

BAO, basilar artery occlusion; FANG-D, visual Field deficit, Aphasia, Neglect, Gaze preference, Dense limb weakness; GCS, Glasgow Coma Scale; NIHSS, National Institutes of Health Stroke Scale.

a Statistical tests: χ^2^ test for categorical variables, *t* test for age, and Mann-Whitney *U* test for other continuous variables, unless otherwise specified.

b One patient with Code Stroke location not documented (inpatient vs emergency department unknown).

c A total of 7 patients were missing NIHSSS (range of GCS scores for these patients was 3-12, median = 5). Four of these patients were from FANG-D screen documented group (range of GCS scores for these patients was 4-13, median = 9) and 3 were from FANG-D screen undocumented group (range of GCS scores for these patients was 3-7, median = 5).

d *P*-values less than .05 signify statistical significance.

Of 121 patients with documented FANG-D screens, 99 were positive, yielding sensitivity of FANG-D to detect a BAO of 81.8% (95% CI: 74.0%-87.7%), which was our primary outcome measure (Tables 2 and 3). FANG-D negative BAO cases had significantly higher GCS scores (15, IQR: 15-15; *P* < .001) and lower NIHSSS (3, IQR: 1-4; *P* < .001) than FANG-D positive cases (13, IQR: 7-15 and 12, IQR: 5-25, respectively). Additional details regarding the 22 FANG-D negative cases (false negatives) are provided in Table S1.

**Table 2:** Comparison of FANG-D positive versus FANG-D negative patients.

	Total (N = 121)	FANG-D negative (n = 22, 18.2%)	FANG-D positive (n = 99, 81.8%)	*P* value^a^
Demographics				
Age, y, Mean ± SD	66 ± 15	62 ± 20	67 ± 14	.2999
Sex (male), n (%)	71 (58.7)	10 (45.5)	61 (61.6)	.1638
Race (White), n (%)	65 (53.7)	15 (68.2)	50 (50.5)	.1326
Inpatient Code Stroke, n (%)	5 (4.1)	1 (4.6)	4 (4.1)	1
Clinical characteristics				
GCS score, median (IQR)	14 (8-15)	15 (15-15)	13 (7-15)	<.001^b^
NIHSS score, median (IQR)	9 (4-23)	3 (1-4)	12 (5-25)	<.001^b^
Radiographic characteristics				
BAO site				
Proximal, n (%)	38 (31.4)	6 (27.3)	32 (32.3)	.6865
Mid, n (%)	43 (35.5)	7 (31.8)	36 (36.4)	
Distal, n (%)	40 (33.1)	9 (41.0)	31 (31.3)	
Totally occlusive BAOs	90 (74.4)	14 (63.6)	76 (76.8)	.2019

BAO, basilar artery occlusion; FANG-D, visual Field deficit, Aphasia, Neglect, Gaze preference, Dense limb weakness; GCS, Glasgow Coma Scale; NIHSSS, National Institutes of Health Stroke Scale Score.

a χ^2^ test for categorical variables, *t* test for age, and Mann-Whitney *U* test for other continuous variables, unless otherwise specified.

b *P*-values less than .05 signify statistical significance.

**Table 3:** Sensitivities of FANG-D screen and NIHSSS among differing BAO populations.

Screen	Population	Screen result	Sensitivity (95% CI)
FANG-D	All BAOs with FANG-D screen^a^ (N = 121)	FANG-D positive (n = 99)	81.8% (74.0%-87.7%)
	Totally occlusive BAOs with a FANG-D screen (N = 90)	FANG-D positive (n = 76)	84.4% (75.6%-90.5%)
	BAOs with FANG-D screen undergoing thrombectomy (N = 42)	FANG-D positive (n = 37)	88.1% (75.0%-94.8%)
NIHSSS ≥ 6	All BAOs with a NIHSSS screen^a^ (N = 197)	NIHSSS ≥ 6 (n = 131)	64.2% (57.4%-70.5%)
	Totally occlusive BAOs with an NIHSSS screen (N = 153)	NIHSSS ≥ 6 (n = 110)	70.1% (62.5%-76.7%)
	BAOs with an NIHSSS screen undergoing thrombectomy (N = 66)	NIHSSS ≥ 6 (n = 46)	68.7% (56.8%-78.5%)

BAO, basilar artery occlusion; FANG-D, visual Field deficit, Aphasia, Neglect, Gaze preference, Dense limb weakness; NIHSSS, National Institutes of Health Stroke Scale Score.

a All BAOs = totally occlusive BAOs + subocclusive BAOs.

Secondary outcomes are listed in Table 3. The sensitivity of a positive FANG-D screen for detecting totally occlusive BAO (N = 90) was 84.4% (95% CI: 75.6%-90.5%), but increased to 88.1% (95% CI: 75.0%-94.8%) for detecting BAO cases that underwent thrombectomy. Among patients with a documented NIHSSS (N = 197), the sensitivity of NIHSSS ≥ 6 for detecting a totally occlusive or subocclusive BAO was 64.2% (95% CI: 57.4%-70.5%), but increased to 70.1% (95% CI: 62.5%-76.7%) for detecting the subset of total occlusive BAOs, and 68.7% (95% CI: 56.8%-78.5%) for detecting BAO cases that underwent emergent thrombectomy.

## 4 Limitations

Our study had several limitations. First, because it was a retrospective study, we could not ensure routine, accurate performance, and documentation of FANG-D screens. Notably, 40% of patients with BAO identified in our study cohort did not have a FANG-D recorded. A plausible reason for some missing scores is that an unexplained comatose-like state is an indication for activating the Code Stroke protocol within our system. Our finding that patients without a FANG-D screen had lower GCS scores supports this reasoning. Another explanation is FANG-D was not routinely collected for inpatient Code Strokes. Second, we only included patients with BAOs for whom a Code Stroke was activated, resulting in their data being entered in the Code Stroke registry. It is possible some patients with acute BAOs evaluated in our health care system during the study period were excluded from our study population. Third, as use of the FANG-D screen represents the standard within our health care system’s EDs, we did not study the specificity of FANG-D for detecting BAO nor did we explore its individual components. Fourth, FANG-D screen and NIHSSS were not routinely performed at the same time, which may lead to variation in the cortical findings for both stroke screens as the patient’s clinical examination evolves. Finally, some patients included in our study had subocclusive lesions, but we specifically cited the sensitivity for patients with total occlusions. Etiologies of posterior circulation stroke include large vessel atherosclerosis leading to subsequent thrombosis or arterial stenosis causing tissue hypoperfusion, which supports including subocclusive BAOs in our study population. Furthermore, some patients with subocclusive lesions undergo thrombectomy as noted in Table S1.

## 5 Discussion

Our report of a large cohort of BAO cases undergoing a qualitative screen consisting of cortical findings typically associated with ACLVO found suboptimal sensitivity for the screen to detect BAO. Based on the lower limit of CIs for sensitivities in Table 3, the FANG-D screen alone has the potential to miss up to one-quarter of patients with any BAO (primary outcome), a totally occlusive BAO, and a BAO undergoing thrombectomy (secondary outcomes). When dealing with a serious, time-sensitive condition, such as BAO, prioritizing high sensitivity is important to ensure most cases are detected to minimize the risk of missing cases that require prompt treatment. An acceptable level of detection for BAO, especially for those patients undergoing thrombectomy, would need to perform above 80% for the lowest CI limit. Although the sensitivity of FANG-D detection for BAO improved for patients undergoing thrombectomy, much of our study period preceded randomized trials demonstrating the benefit of thrombectomy for BAO. Only recently have expert panel recommendations for thrombectomy been proposed in the setting of BAO.^14^ It is notable that for 5 of 22 patients with false negative FANG-D screens, their NIHSSS was consistent with positive FANG-D screen elements (eg, partial hemianopia, partial gaze palsy, and mild aphasia noted in Table S1). As the FANG-D screen and NIHSSS were not performed contemporaneously, it may be that findings present on the NIHSSS were not present during the initial physician screen, or findings noted on the NIHSSS were missed by the EM or inpatient physician on the initial screen.

Occlusions of the proximal or middle segments of the basilar artery usually result in large pontine strokes with either hemiplegia or quadriplegia,^1^ which should result in the FANG-D screen being positive because it includes the finding of significant weakness in any limb. Locked-in syndrome, or ventral pontine syndrome, occurs in patients with compromised blood flow to the inferior pons and may have clinical features such as quadriplegia with preservation of vertical eye movement, blinking, and consciousness.^15^ However, a significant minority of patients experiencing a BAO present with locked-in syndrome, with estimates ranging from around 10% to 15% of cases.^16^ Nonetheless, physicians should always consider acute BAO in patients presenting with a sudden, otherwise unexplained comatose-like state and promptly investigate with CTA. In our study cohort, patients without a FANG-D screen had significantly lower GCS scores than those who did, which is consistent with our criterion of Code Stroke activation in sudden, unexplained comatose-like states. Other findings of proximal and mid BAO include reduced consciousness, bilateral extensor plantar sign, dysarthria, dysphagia, horizontal gaze paresis, and other cranial nerve palsies.^1^ As NIHSSS does not include a detailed assessment of cranial nerves, this may contribute to its poor sensitivity in detecting BAO in this region.

As BAO represents a minority of LVO strokes, there are sparse data on the performance of traditional stroke and LVO screens to identify BAO. Nonetheless, it is known that many routinely used stroke identification screens, such as the face arm speech test (FAST), are less sensitive for detecting posterior circulation strokes than those involving the anterior circulation.^17^ This can be problematic for detecting BAO as, eg, the American Stroke Association/American Heart Association stroke severity screen recommends performing the FAST first and not proceeding with an LVO screen in patients who screen negative for FAST.^18^ A similar limitation applies to the Recognition of Stroke in the Emergency Room (Rosier) scale, which can miss up to 50% of posterior circulation strokes.^19^ The performance of other stroke identification and LVO screens (eg, Los Angeles Prehospital Stroke Screen, Los Angeles Motor Scale, Field Assessment Stroke Triage for Emergency Destination, Rapid Arterial Occlusion Evaluation, and Vision Aphasia Neglect) for BAO detection is unknown because the diagnostic studies of these screens excluded BAO in their LVO definitions.^6^^,^^12^^,^^20^, ^21^, ^22^

In our secondary outcome analysis, the FANG-D screen performed better than an NIHSSS threshold ≥ 6 for detecting all categories of BAO (Table 3). Although an NIHSSS <6 suggests a mild stroke, it is recognized that NIHSSS is weighted toward anterior circulation findings.^23^ As an example, the NIHSSS does not include an assessment of gait or truncal ataxia, which can be present in posterior circulation strokes and may be the only disabling neurologic deficit present.^24^ For this reason, activating a Code Stroke solely based on an NIHSSS threshold risks potential exclusion of patients with BAO. As Braksick and Rabinstein^25^ have pointed out, neurologic examination should be expanded beyond the NIHSSS assessment to include assessing cranial nerves and gait, which are both inadequately evaluated by NIHSSS. Nonetheless, it must be acknowledged that patients with NIHSSS <6 were excluded from the recently positive BAOCHE thrombectomy trial in BAO, and patients with NIHSSS <10 were excluded from the ATTENTION trial.^4^^,^^5^ These exclusions highlight the need to further study the benefit of thrombectomy for BAO in patients with NIHSSS below these thresholds or who have otherwise been excluded from BAO thrombectomy trials.^14^^,^^26^

The basilar artery and its branches supply oxygenated blood to areas in the posterior circulation including the brainstem and cerebellum. Although posterior circulation strokes include BAO, many posterior circulation strokes do not involve a BAO. The posterior circulation includes the extracranial and intracranial vertebral arteries, the basilar artery, posterior cerebral arteries and their branches, with the left- and right-vertebral arteries joining to form the basilar artery at the pontomedullary junction. In the setting of a BAO, collateral flow from the carotid system via the posterior communicating arteries can provide perfusion to the posterior cerebral arteries and penetrating vessels emanating from the distal basilar, which contributes to the varied clinical signs that can be associated with BAO. This collateral flow may explain why a visual Field deficit does not accompany many BAOs, as the occipital lobes and other cortical areas benefit from collateral circulation provided by the circle of Willis. However, when the temporal and occipital lobes are involved due to occlusion at the top of the basilar artery, patients can develop cortical blindness, hemianopia, amnesia, and agitation.^1^ Top of the basilar artery syndrome occurs when the most distal aspect of the basilar artery is occluded leading to infarction of the midbrain and superior cerebellum. Clinical characteristics include somnolence, confusion, hallucinations, and visual-oculomotor deficits, but generally do not include limb motor dysfunction.^27^^,^^28^ Some distal BAO strokes involve the midbrain and thalamus, manifesting with decreased consciousness, quadriparesis, and nuclear or supranuclear oculomotor dysfunctions.^1^

The varied clinical presentations described above demonstrate the challenge of relying on stroke severity and LVO screens comprising cortical findings to detect BAO. Our results suggest a qualitative screen composed of cortical signs alone and an NIHSSS ≥ 6 both lack sufficient sensitivity to be used to detect an acute BAO, including totally occlusive ones that undergo acute thrombectomy. Now that mechanical thrombectomy has been shown to be beneficial for BAO, prospective studies are needed to assess qualitative and quantitative LVO screens to detect BAO.

## Author Contributions

CA, RRK, and AWA conceived the study and designed the project. CA, RRK, DS, TWW, SS, HY, JBR, GD, JDC, KJT, and AWA contributed to data collection, analysis, and interpretation. CA and AWA drafted the manuscript, and all authors contributed to the editing and revision of the final version of manuscript. CA and AWA take full responsibility for the final version of the paper.

## Funding and Support

By *JACEP Open* policy, all authors are required to disclose any and all commercial, financial, and other relationships in any way related to the subject of this article as per ICMJE conflict of interest guidelines (see www.icmje.org). The authors have stated that no such relationships exist.

## Conflict of Interest

All authors have affirmed they have no conflicts of interest to declare.
