# Supplementary material for: Sensitivity of a Qualitative 5-Element Cortical Sign Screen for Detecting Acute Basilar Artery Occlusion
Source: J Am Coll Emerg Physicians Open. 2025 May 21;6(4):100167. doi: 10.1016/j.acepjo.2025.100167 (PMC12149534; doi:10.1016/j.acepjo.2025.100167)
Supplement: Supplementary Table [file mmc1.docx]

**Supplemental Table 1: Characteristics of false negative FANG-D patients (N = 22)**

| **Age** | **Sex** | **GCS Score** | **NIHSSS**  **Score** | **Positive NIHSSS Elements** | **Deficits Noted on Provider Exam** | **BAO site** | **Occlusion Type** | **DWMRI positive brain territories** | **Thrombectomy Performed** | **Discharge**  **mRS** |
| --- | --- | --- | --- | --- | --- | --- | --- | --- | --- | --- |
| 86 | F | 11 | 1 | - Dysarthria - Mild to moderate | Dysarthria, nausea, vomiting | Mid | Occlusive | - Right frontal lobe - Right occipital lobe | Y | 2 |
| 71 | F | 12 | 9 | - LOC – Drowsy - Facial Palsy – Minor - Left Arm Motor – Drift - Right Arm Motor – Drift - Left Leg Motor – Drift - Right Leg Motor – Drift - Limb Ataxia - Present in two limbs - Dysarthria - Mild to moderate | Dysarthria, nausea, vomiting, left upper extremity weakness, confusion | Dist | Occlusive | - Left occipital lobe - Bilateral cerebellar hemispheres - Cerebellar vermis | N | 4 |
| 68 | M | 15 | 4 | - Facial Palsy – Minor - Limb Ataxia - Present in one limb - Sensory - Partial loss - Dysarthria - Mild to moderate | Vertigo, left-sided facial paresthesias, left facial droop, slurred speech | Prox | Sub-occlusive | Negative | N | 0 |
| 69 | M | 15 | 4 | - Best Gaze - Partial gaze palsy - Limb Ataxia- Present in two limbs - Dysarthria - Mild to moderate | Dysarthria, blurred vision, abnormal finger-to-nose | Mid | Occlusive | - Left cerebral peduncle - Left dorsal tectum - Right central pons - Left cerebellar hemisphere | Y | 1 |
| 41 | M | 15 | 6 | - Visual – Partial hemianopia - Facial Palsy – Partial - Limb Ataxia - Present in two limbs Sensory – Partial loss | Bidirectional nystagmus, right- sided facial asymmetry, abnormal finger to nose and heel to shin on left, ataxia | Mid | Occlusive | - Left lateral pons - Right cerebellar hemisphere - Cerebellar vermis - Left cerebellar tonsil | N | 2 |
| 46 | M | 15 | 3 | - Facial Palsy – Minor - Sensory - Partial loss - Dysarthria - Mild to moderate | Vertigo, dysarthria, nausea, decreased sensation left upper extremity, decreased sensation left lower extremity | Prox | Occlusive | - Right cerebellar tonsil - Right cerebellar hemisphere | N | 1 |
| 68 | F | 15 | 2 | - Best Gaze - Partial gaze palsy - Limb Ataxia - Present in one limb | Left upper extremity weakness, dysarthria, left facial droop | Dist | Sub-occlusive | - Right anterolateral thalamus - Right medial midbrain - Left cerebellar hemisphere - Cerebellar vermis - Right cerebellar hemisphere | N | 0 |
| 69 | M | 8 | 0 | None | Blurry vision, dizziness, nausea, vomiting | Mid | Sub-occlusive | - Left superior cerebellar peduncle | Y | 0 |
| 30 | F | 15 | 7 | - Facial Palsy – Minor - Left Arm Motor – Drift - Left Leg Motor – Drift - Limb Ataxia - Present in two limbs - Sensory - Partial loss - Dysarthria - Mild to moderate | Vertigo, nausea, vomiting left facial droop, left upper extremity weakness, abnormal finger-to-nose, decreased left upper extremity sensation, decreased left lower extremity sensation, gait ataxia | Prox | Occlusive | - Posterior right frontal lobe - Right parietal lobe - Right cerebellar hemisphere - Left lateral medulla | N | 6 |
| 89 | F | 13 | 3 | - LOC Questions - Answers one correctly - Facial Palsy - Partial | Vertigo | Prox | Occlusive | - Bilateral cerebellar hemispheres | N | 3 |
| 85 | M | 15 | 0 | None | Dizziness, right upper extremity paresthesias | Prox | Occlusive | Negative | N | 0 |
| 76 | F | 15 | 4 | - Facial Palsy – Minor - Left Leg Motor – Drift - Limb Ataxia - Present in one limb - Dysarthria - Mild to moderate | Dizziness, ataxia, left lower extremity weakness, nystagmus, facial asymmetry, left upper extremity dysmetria | Dist | Sub-occlusive | - Left cerebellar hemisphere, - Minimal right cerebellar hemisphere | Y | 3 |
| 70 | F | 15 | 2 | - Sensory - Partial loss - Dysarthria - Mild to moderate dysarthria | Dysarthria, left facial paresthesias | Mid | Occlusive | - Left paramedian pons | N | 4 |
| 87 | F | 15 | 5 | - Left Leg Motor – Drift - Sensory - Partial loss - Best Language - Mild to moderate aphasia - Limb Ataxia - Present in two limbs | Dysarthria, dizziness | Dist | Occlusive | - Bilateral cerebellar hemispheres | Y | 2 |
| 73 | F | 15 | 2 | - Sensory - Partial loss - Dysarthria - Mild to moderate dysarthria | Vertigo, dysarthria, nausea, vomiting, down and out left gaze palsy | Dist | Occlusive | - Right paramedian pons | N | 1 |
| 36 | F | 15 | 5 | - Best Gaze - Partial gaze palsy - Facial Palsy – Minor - Left Arm Motor – Drift - Left Leg Motor – Drift - Dysarthria - Mild to moderate | Dizziness, disconjugate gaze | Dist | Sub-occlusive | - Left cerebellar hemisphere | N | 3 |
| 75 | M | 15 | 4 | - Left Arm Motor – Drift - Limb Ataxia - Present in two limbs - Dysarthria - Mild to moderate dysarthria | Left upper extremity weakness | Dist | Occlusive | - Right frontal lobe - Left parietal lobe - Posterior left temporal lobe - Bilateral cerebellar hemispheres | N | 2 |
| 44 | F | 15 | 0 | None | Sixth nerve palsy, Tinnitus, Syncope | Dist | Sub-occlusive | Negative | N | 0 |
| 28 | M | 13 | 0 | None | Vertigo, left arm parasthesias, left face parasthesias | Mid | Sub-occlusive | Not performed | N | 1 |
| 49 | F | 15 | 0 | None | Vertigo and nausea | Mid | Sub-occlusive | Negative | N | 2 |
| 75 | M | 15 | 1 | - Limb Ataxia - Present in one limb | Aphasia, ataxia | Prox | Occlusive | - Right posterior insula and temporal operculum - Left occipital lobe | N | Not recorded |
| 38 | M | 15 | 2 | - Sensory - Partial loss - Dysarthria - Mild to moderate dysarthria | Nausea, paresthesias of the lips and double vision, right facial-droop, dysarthria; truncal ataxia, abnormal finger-to-nose | Dist | Occlusive | - Bilateral cerebellar hemispheres | Y | 1 |

Abbreviations: GCS = Glasgow Coma Scale, NIHSSS = National Institutes of Health Stroke Scale Score, BAO = basilar artery occlusion, DWMRI = diffusion-weighted magnetic resonance imaging, mRS = Modified Rankin Score, Prox = Proximal, Dist = Distal
